# Spatial and Temporal Dynamics of Attentional Guidance during Inefficient Visual Search

**DOI:** 10.1371/journal.pone.0002219

**Published:** 2008-05-21

**Authors:** Alexandre Zenon, Suliann Ben Hamed, Jean-René Duhamel, Etienne Olivier

**Affiliations:** 1 Laboratory of Neurophysiology, Université catholique de Louvain, Bruxelles, Belgium; 2 Institut des Sciences Cognitives, CNRS, Bron, France; University of Granada, Spain

## Abstract

Spotting a prey or a predator is crucial in the natural environment and relies on the ability to extract quickly pertinent visual information. The experimental counterpart of this behavior is visual search (VS) where subjects have to identify a target amongst several distractors. In difficult VS tasks, it has been found that the reaction time (RT) is influenced by salience factors, such as the target-distractor similarity, and this finding is usually regarded as evidence for a guidance of attention by preattentive mechanisms. However, the use of RT measurements, a parameter which depends on multiple factors, allows only very indirect inferences about the underlying attentional mechanisms. The purpose of the present study was to determine the influence of salience factors on attentional guidance during VS, by measuring directly attentional allocation. We studied attention allocation by using a dual covert VS task in subjects who had 1) to detect a target amongst different items and 2) to report letters briefly flashed inside those items at different delays. As predicted, we showed that parallel processes guide attention towards the most relevant item by virtue of both goal-directed and stimulus-driven factors, and we demonstrated that this attentional selection is a prerequisite for target detection. In addition, we show that when the target is characterized by two features (conjunction VS), the goal-directed effects of both features are initially combined into a unique salience value, but at a later stage, grouping phenomena interact with the salience computation, and lead to the selection of a whole group of items. These results, in line with Guided Search Theory, show that efficient and rapid preattentive processes guide attention towards the most salient item, allowing to reduce the number of attentional shifts needed to find the target.

## Introduction

A rapid identification of pertinent stimuli is crucial in the natural environment and this ability relies on a selection mechanism known as spatial attention, which permits the allocation of more neural resources to process behaviorally significant elements. Our understanding of attentional mechanisms mainly comes from studies using covert visual search (CVS) tasks – where subjects have to detect a given target amongst distractors, without performing eye movements. Two types of CVS are classically distinguished: 1) efficient CVS in which the reaction time (RT) remains constant irrespective of the number of items, suggesting that visual information is processed in parallel, and 2) inefficient CVS in which RT increases proportionally to the number of items, a relationship known as the “set-size effect” and classically regarded as evidence for a serial processing of visual information. However, in inefficient CVS, the slope of the regression line between RT and the number of CVS items typically varies from 25 to 60 ms/item, leaving very little time to process each item serially [1]. Moreover, estimates of the attentional dwell time range between 200 and 400 ms, values which are incompatible with the attentional shift time estimated from the set-size slope [2], [3]. This suggests that, besides serial mechanisms, parallel mechanisms most likely contribute to visual information processing in inefficient CVS [4], [5]. The Guided Search Theory (GST) proposed that the role of preattentive parallel mechanisms in visual search is to guide attention on the basis of the salience value assigned to each CVS item [5]–[7]; this salience value is supposed to be assessed from both goal-directed (or top-down) and stimulus-driven (or bottom-up) factors.

Importantly, the conclusion of most studies on CVS relied on the interpretation of changes in RT and set-size slope in different experimental conditions. However, because variations in RT may be caused by different sensory, sensori-motor and motor factors, it is always difficult to interpret them in terms of attention allocation. Indeed, several recent studies have shown that it is possible to explain the variation of performance as a function of set-size during CVS without requiring serial attentional mechanisms (e.g. see [8]–[10]). Therefore, in order to study thoroughly attentional allocation during CVS, it is necessary to demonstrate a spatially specific perceptual improvement during CVS, revealing the attentional selection of one – or multiple – item(s).

Several studies have already used this approach. For example, Maljkovic and Nakayama [11], [12], were able to demonstrate, in a pop-out visual search task, that attention systematically shifted onto the target. These results were extended to inefficient CVS by Nothdurft [13], who used the illusory line motion effect to probe attentional allocation on the target. Kim & Cave [14], went a step further by studying the attentional allocation during CVS, not only on the target, but also on distractors. These authors probed attentional allocation by using both a dot detection task and a letter detection task. This study showed, notably, that in conjunction CVS, where two features (shape and color) define the target, letters located inside items sharing one feature with the target were detected more often, suggesting the existence of a preferential selection of potentially pertinent items. However, in this study, attentional guidance was affected by strong Gestalt grouping mechanisms [15] making the interpretation of these results in terms of attentional guidance difficult. Moreover, Kim & Cave did not investigate the role of stimulus-driven salience, known to play a central role in GST. Finally, in order to avoid saccades towards the CVS items, Kim & Cave used very short presentation times, and, consequently, the search process was most likely conducted in visual memory instead of on visually available stimuli [16].

The purpose of the present study was to extend the Kim & Cave study in order to 1) determine the magnitude, and the timing of the effect of both goal-directed and stimulus-driven salience factors, 2) determine whether GST and/or grouping phenomena are responsible for the preferential selection of items sharing a feature with the target.

To address these issues, we conducted three experiments using a dual CVS task, in which a letter detection task was used to probe attention allocation during the performance of a difficult CVS where the distractors could have different levels of resemblance with the target. We used long presentation times to allow attention to select visually available items and eye fixation was controlled online during the task performance.

**Figure 1:**
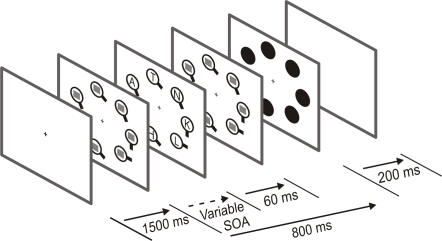
Experimental design of Experiment 1. A fixation point was displayed for 1500 ms, followed by the presentation of 7 CVS items. The target was the circle with a vertical line. After a variable delay, the mask displayed inside each CVS item was replaced, for 60 ms, by letters. Eight hundred ms after the onset of the CVS item display, CVS items were replaced by black disks flashed for 200 ms.

In Experiment 1, we determined the influence of goal-directed, stimulus-driven and spatial factors on attentional allocation during an inefficient feature CVS task in which subjects had to detect a vertically oriented item displayed amongst distractors of various, non-vertical orientations. Letters were also displayed inside these items at different intervals with respect to VS items onset (see Fig. 1). The influence of goal-directed salience factors was determined by analyzing the letter detection probability in each CVS item as a function of similarity with the target. We predicted that letters located either inside the target or inside items resembling the target, should be detected more often. To study the influence of stimulus-driven factors, we also analyzed the effect of orientation contrast, i.e. the difference in orientation between neighboring items, and our prediction was that letter detection should be better for greater orientation differences between adjacent items. Finally, we investigated the potential effect of spatial location of the CVS items on attentional allocation by analyzing letter detection as a function of their position in the display, since it has been suggested that letters located in the inferior hemifield should be detected more often [17], [18].

Whereas Experiment 1 will allow us to investigate attentional allocation during CVS, it cannot help us to determine whether attentional selection of the target is a consequence of, or a prerequisite for, the target detection. To address this issue, in Experiment 2, we used the same dual task as in Experiment 1 but the duration of the CVS item presentation was changed across trials and the letters were always flashed at the end of the trial. We predicted that if attention selection is critical to target detection, there should be a tight correlation between letter detection performance in the target and target detection probability. Specifically, we expected to find that target detection should be at chance level in the absence of attentional selection (i.e. 0% letter detection) and that the slope of the target detection function should link minimal and maximal performance levels of both variables.

Finally, in Experiment 3, we used conjunction search to assess the role of grouping phenomena in the selection of the items sharing a feature with the target. Kim & Cave [14] showed that, in conjunction search tasks where the target was defined by color and shape, subjects tended to detect more often the letters that were displayed inside items that shared one feature (either color or shape) with the target than in the other distractors. The first interpretation of these results, in line with GST, assumes that each feature (color and shape in this case) is encoded into a separate feature map, where the activity induced by each item depends on its resemblance with the target with respect to this feature. Then, the two feature maps corresponding to color and shape are added together to lead to a global activation map. The items that do not share any feature with the target will induce the minimum activation in this map, the items sharing one target feature will induce intermediate activation, and the target will generate the maximum activation, resulting from the addition of the maximum activation values in the two feature maps. The second interpretation of the Kim & Cave results is that color and shape, being in this case both very easily discriminable (green versus red and squares versus circles) induce strong gestalt grouping effects [15], [19], [20]. Therefore, the group of items sharing one target feature would be selected as a whole, and the target would be searched only in this group [21], [22]. To distinguish between these two hypotheses, we studied attentional allocation during a conjunction CVS (orientation and color), where three different levels of orientation difference between target and distractors were used (see Fig. 3). Again, a letter detection task was used to probe attention during the CVS task, as in Experiments 1 and 2. In order to induce grouping effects, we used only two easily discriminable color levels. Our predictions were that, if GST is correct, the salience effect of color and orientation should have an independent (or additive) effect on letter detection, whereas if the grouping hypothesis is correct, we should find an interaction between both effects: orientation should affect detection probability of the letters in the items sharing the target color, whereas items with the non-target color should be all excluded from the search process, and hence, their selection probability should not be affected by their orientation [21].

Additionally, to have a more precise idea of the timing of these effects, we modified the letter display, allowing us to monitor attentional allocation with a higher temporal resolution. To achieve this increased resolution, instead of displaying a letter inside all VS items at the same time, a single letter was displayed in a given item at a given time, and this letter was moved and changed every 45 ms.

## Materials and Methods

### Experiment 1

#### Subjects

Twelve healthy subjects (21–25 years old), with normal, or corrected to normal, vision participated in Experiment 1; all subjects were native French speakers. All experimental procedures were approved by the Ethics Committee of the Université catholique de Louvain, and subjects gave their written informed consent.

#### Task

In this experiment, we used a probe paradigm [14] in which subjects had to report the presence, or absence, of a target displayed amongst 7 items arranged in a circle around a central fixation point (see Fig. 1). In addition, subjects had to identify letters flashed for 60 ms inside each CVS item, at different stimulus onset asynchronies (SOAs) with respect to the onset of the CVS item presentation. The rationale behind this paradigm is that the letter detection probability should be higher at positions where attention is located, allowing us to infer a posteriori attentional allocation in both space and time.

Before each experiment, subjects were trained in the CVS task alone, then on the letter identification task alone and finally they practiced the dual task. At the end of the training session, a staircase procedure was used to adjust the contrast of the CVS items against the background in order to make sure that all subjects achieved a performance of 70 % of correct trials in the CVS task. The final contrast obtained was, on average, equal to 70 %. The gray level-luminance function of the screen was obtained with a psychophysical method of gamma correction (Bordier C. & Knoblauch K., personal communication).

At the beginning of each trial, a fixation point was presented at the center of a computer screen situated at a distance of 55 cm, in front of the subjects; the subjects had to fixate this point throughout the task. The eye position was monitored using an electro-oculogram and trials were interrupted, and repeated, whenever subjects broke fixation. After a 1500 ms delay, seven grey circles (1.8 degrees wide) were displayed equidistantly at an eccentricity of 4 deg. These CVS items were filled with a white noise mask and had a small bar attached to them, that could be either vertical (target) or oriented at 30, 60 or 90 degrees from the vertical (distractors). The target was present in 50 % of the trials and at least two distractors of each type were present in each trial. After a variable SOA (50, 300, 500 or 700 ms), the masks displayed inside the circles were replaced by letters (1.1 degree wide) which were flashed for 60 ms and then replaced by a new mask. In a given trial, every letter was different. Eight hundred ms after the onset of CVS item presentation, all items were replaced by black disks displayed for 200 ms.

Subjects were instructed to press, as fast as possible, the « one » key of the numeral pad of a computer keyboard to indicate that the target was present or the « three » key to indicate it was absent; then they had to type on the keyboard, without any time constraint, the letters they identified inside the CVS items. Subjects then triggered the next trial by pressing the space bar. The experiment was divided in 5 blocks of 160 trials each.

#### Control task A: letter detection only

In order to determine whether the effects we observed in the main experiment were specific to CVS, and not to the letter detection task, a control experiment was performed under the same experimental conditions as in Experiment 1, except that subjects (n = 10) were only asked to report as many letters as possible without taking notice of the CVS items.

#### Control task B: target detection only

This second control experiment was identical to the main experiment (Experiment 1) except that subjects (n = 7) only had to report the presence, or absence, of the CVS target, without paying attention to the letters. In this experiment, the number of CVS items was either 4 or 8 in order to allow us to determine the difficulty of the CVS task and to estimate the “set-size effect”.

#### Data analysis

A goal-directed salience value, varying from 4 to 1, was assigned to each CVS item depending on its similarity with the target, 4 being attributed to the target and 1 to the most dissimilar distractor. In addition, an orientation contrast index was calculated for each CVS item by computing the sum of the differences between the orientation of the bar attached to the item and its two neighbors. For example, an item with a 30° oriented bar (salience value: 3) surrounded by the target (salience: 4) and by an item with a 90° oriented bar (salience: 1) had an orientation contrast index of 3 (|3−4|+|3−1|).

#### Statistical analysis

We used ANOVAs on repeated measurements (RM ANOVA) for most statistical analyses. In order to achieve normality, percentages of detection were arcsine-root transformed, a transformation appropriate for data that lie between an upper and lower bound [23]. When data distribution of probability differed from normality, a non-parametric ANOVA on ranks test was performed.

### Experiment 2

**Figure 2:**
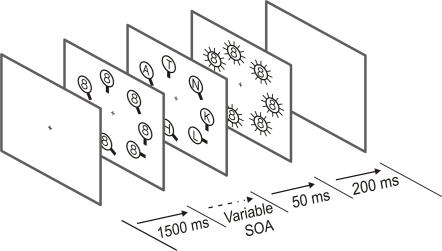
Experimental design of Experiment 2. Only the case where two targets were displayed simultaneously is illustrated here. Compared to Experiment 1, the CVS items display duration was variable (50%, 75% and 90% correct response in CVS), and letters were always displayed during the last 50 ms of the trial.

Eight subjects (20–29 years old) participated in Experiment 2, which was exactly the same as Experiment 1 except that 1) the duration of the CVS item presentation varied from trials to trials, and 2) we used a different masking stimulus, the digit 8, which has proved to be more efficient as a mask in a letter identification task (see Fig. 2) [24]. The duration of the CVS item presentation was determined for each subject at the beginning of the experimental session by means of a staircase procedure adjusted to obtain 50, 75 or 90% of correct responses in the CVS task, while the contrast was kept constant (70%). In this staircase procedure, subjects had to perform the CVS task only, without reporting the letters displayed inside the CVS items. The duration of CVS items presentation was then adjusted to these three values in the main experiment and the three display durations were counterbalanced across trials. In addition, also in contrast to Experiment 1, the letters were always flashed during the last 50 ms of the CVS item presentation. Then, all stimuli were replaced by a mask: the letters were masked with a figure-eight and the CVS items were masked with circles with 12 bars attached every 30°, from 0° to 330°. One, two or no target could be displayed, these three conditions being equally probable (33% each). In the foregoing analyses, we only considered trials where one or no target was displayed. The response keys used to indicate that the target was present or absent were the same as in Experiment 1. The subjects were not informed that two targets could appear simultaneously in some trials and the response required in these cases was the same as when only one target was detected. As in Experiment 1, subjects had also to type the detected letters, with no temporal constraint and the next trial was triggered by pressing the space bar. In this experiment, eye movements were monitored by using an infrared camera (Thomas Recording).

### Experiment 3

Eight subjects participated in Experiment 3. The task was, as in Experiments 1 and 2, to detect the presence of the target (50% probability), as quickly as possible, and to report as many letters as possible. Eye position was controlled by means of an infrared eyetracker and trials were interrupted, and repeated, whenever subjects broke fixation. In this task, three blue and three yellow isoluminant circles were displayed on the screen, at fixed eccentricity (4°). The orientation of the bar attached to them was 0°, 20° or 40° from the vertical. The target was defined as the blue circle with a vertically oriented bar. In target present trials, each item of a given color had a different orientation. In target absent trials, two items with the target color had a 20° orientation.

**Figure 3:**
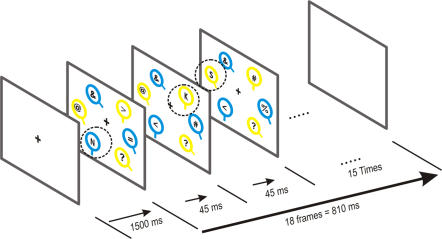
Experimental design of Experiment 3. The target was the blue circle with a vertical line. Every 45 ms, a different letter was displayed in a different location (surrounded by dotted circles, not shown on actual display). All other items contained irrelevant signs.

One ASCII sign was displayed inside each VS item but, in only one item, this sign was a letter; in the other VS items, the signs were irrelevant (‘&’, ‘%’, etc…). At every frame change (i.e. every 45 ms), the relevant letter and two irrelevant signs were changed; the new relevant letter was flashed at another location (see Fig. 3). The duration of the CVS items display was 810 ms, corresponding to 18 frame changes. The letter was displayed in each VS item in a pseudo-randomized order so that, in a given trial, a different relevant sign appeared three times inside each VS item (n = 6), with a variable delay between each presentation. In the analyses, in order to have a sufficient number of trials in each condition, we grouped adjacent letter onset times by pairs, yielding 9 different letter onset time values (1–45 ms, 90–135 ms, 180–225 ms, …). An additional control experiment was conducted in which the display was exactly the same, but subjects only had to report the letters.

## Results

### Experiment 1

#### General performance

We first found that the CVS task used in the present study was inefficient. The control task B (see Methods) showed that RTs increased as a function of the number of CVS items (2-way ANOVA RM : F = 8.12, p = 0.029) and were larger when the target was absent than when present (F = 10.19, p = 0.019). The difference between RT in the 8-items CVS task (median±interquartile interval (IQI) : 1378±673 ms for target absent and 863±666 ms for target present) and the 4-items CVS task (1172±590 ms for target absent and 805±555 ms for target present) was 97±237 ms (median±IQI) for target absent trials and 69±109 ms for target present trials. This corresponds to a mean increase of 20 ms per item, a value compatible with the magnitude of set-size effects classically reported in the literature for inefficient CVS tasks [1].

In the main experiment (Experiment 1), we found that the number of detected letters increased with SOA. On average, 0.8, 0.97, 1.18, 1.39 letters were reported for 50, 300, 500 and 700 ms SOAs , respectively. Median RT in the CVS task was 1190 ms (IQI = 282 ms, n = 12) and longer SOAs for letter display, were associated with longer RTs in the CVS task. This finding can be explained if we assume that the processing of the letters and their storage in memory occurred during the preparation of the motor response of the CVS task, leading to an interference. For the CVS task, when the target was present, we found 75.2±13.6% of HITS (correct detection of the target) and 24.8±13.6% of MISSES (failure to detect the target when present); when the target was absent, we observed 81.0±13.6% of CORRECT REJECTIONS (correct rejection of target presence) and 19.0±13.6% of FALSE ALARMS (detection of the target while absent).

#### Influence of goal-directed salience on attentional allocation

**Figure 4:**
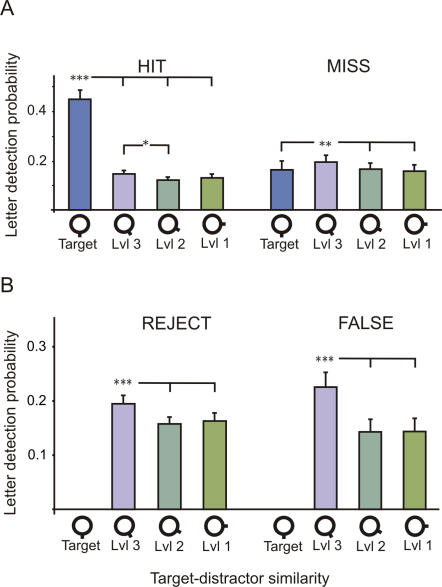
Effect of target-distractor similarity on letter detection performance. A. Main effect of target-distractor similarity on “target present” trials for HIT (left) and MISS (right) trials. In the HIT trials, letters located inside the target were more likely to be detected than other letters, whereas this effect was reversed in the MISS trials. In both types of trials, letters located in level 3 distractors were more often detected than those located in level 2 and level 1 distractors (*: p<0.01; **: p<0.001; ***: p<0.0001). Y axis: percentage of letter detection. X axis: target-distractor similarity varying from 4 (target) to 1 (most dissimilar distractor). Error bars correspond to standard errors. B. Main effect of target-distractor similarity on “target absent” trials for REJECT (left) and FALSE ALARM (right) trials. In both types of trials, letters located in level 3 distractors were more likely detected than those located in level 2 and level 1 distractors. Same conventions as in A.

We first investigated the influence of the goal-directed salience factor (similarity with the target) on the probability to detect letters inside CVS items. For all types of responses (HITS, MISSES, CORRECT REJECTIONS and FALSE ALARMS), we found that the target-distractor similarity significantly influenced letter detection probability (main effect: F ranged from 5 to 280, all p<0.02). For HIT responses, the probability to detect a letter was higher when flashed inside the target (post-hoc: all p<0.0001) and, to a lesser extent, inside level 3 distractors, i.e. the most resembling distractors (post-hoc: p = 0.03, Fig. 4A). For MISS responses, the letter detection rate was higher in the level 3 distractors than in the target (post-hoc: p = 0.008). When the target was absent (CORRECT REJECTION and FALSE ALARM), letters flashed inside the level 3 distractors were more likely to be detected than those flashed inside level 2 and 1 distractors (post-hoc: all p<0.0001, Fig. 4B).

**Figure 5:**
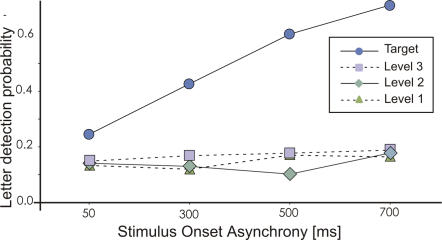
Target-distractor similarity - SOA interaction for HIT trials. The benefit of letter detection inside the target for HIT trials was already significant for a 50 ms SOA and it increased monotonically with the SOA. The detection probability is represented on the Y-axis, SOAs on the X-axis.

In addition, we investigated the influence of the SOA (delay between the presentation of CVS items and the letter display) on the probability of letter detection. For HIT trials, we found a significant interaction between target-distractor similarity and soa (F = 49,90, p<0.0001). This interaction can be explained by the fact that, although the detection rate of letters flashed inside the distractors was not influenced by the SOA, the detection rate of letters flashed inside the target increased with the SOA (post-hoc: all p<0.0001, Fig. 5). It is noteworthy that, even for SOA as short as 50 ms, the detection rate of letters flashed inside the target was already significantly higher than for letters flashed inside distractors.

In order to determine whether these effects were actually goal-directed, we conducted a control experiment where subjects only had to detect the letters, without paying attention to the CVS items (control task A, see Methods). In this task, we failed to found an influence of the target-distractor similarity on the letter detection (F = .03, p = .99).

#### Influence of stimulus-driven factors and spatial location on attentional allocation

**Figure 6:**
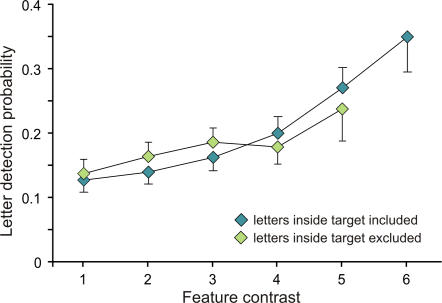
Effect of local orientation contrast on letter detection probability. The letter detection rate increased proportionally with the orientation contrast. Contrast level is represented on the X-axis and percentage of detection on the Y-axis. Error bars correspond to standard errors.

We then investigated the influence of a stimulus-driven salience factor (the local orientation contrast) on the target and letter detection rates. Orientation contrast was computed for each item by estimating its dissimilarity with its two neighbors (see Methods). As shown in Figure 6, the letter detection probability increased proportionally with the local orientation contrast (ANOVA on Ranks, chi-square = 52.81, p<0.001; post-hoc: all pairwise multiple comparisons significant except contrast 6 vs 5). However, it may be argued that this effect was due to the higher detection rate of letters displayed inside the target because, on average, the target had a higher orientation contrast value than the distractors. In order to rule out this hypothesis, we performed the same analysis after removing all letters detected inside the target and we found identical results i.e. an increased detection probability of letters as a function of the local orientation contrast (ANOVA on ranks, chi-square = 39.07, p<0.001, Fig. 6). In addition to the effect on letter detection, we found that the local orientation contrast also influenced the target detection probability (RM ANOVA F = 5.26, p = 0.001). However, this effect was essentially due to a lower detection rate of the target when surrounded by two distractors very similar to the target (post-hoc: p<0.001).

**Figure 7:**
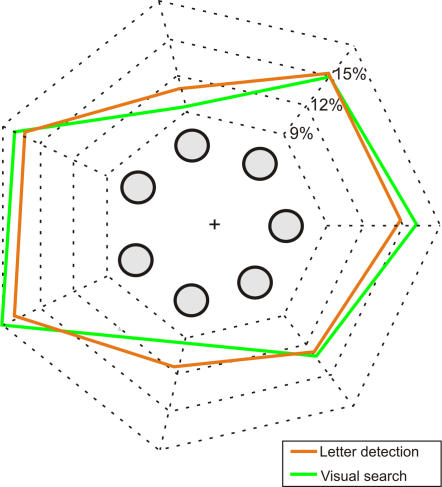
Effect of position. Radial plot showing the proportion of objects detected in each position on the display for visual search (green) and letter detection (orange) in the dual task. CVS items and letters located on the horizontal meridian were detected more often. Item positions on the graph correspond to the positions in the display as seen by subjects.

As far as the effect of spatial location on attentional allocation was concerned, we found a significant effect of the item position in the display on both the target (F = 6.32, p<0.0001) and letter detection (F = 18.3, p<0.0001) rates; namely a higher detection rate was observed for CVS items and letters located near the horizontal meridian (Figure 7).

In order to determine whether the effect of orientation contrast and spatial location on letter detection probability was specific to CVS, we analyzed letter detection performance in the control task A, where subjects were only asked to report letters without performing the CVS task. We confirmed a significant effect of position (F = 2.36, p = .045) with the same preference for horizontal positions as in Experiment 1, but we failed to find an effect of the orientation contrast (F = 1.04, p = .40). This finding indicates that the CVS items have to be processed in order to induce a local orientation contrast effect, suggesting that the stimulus-driven effect of the orientation contrast is influenced by the task at hand. In contrast, the significant effect of position for the letter detection performed alone was expected since, if attentional allocation probability depends on spatial location, it will affect stimulus detection irrespective of the nature of the stimulus to be detected.

### Experiment 2

The results of Experiment 2 confirmed those of Experiment 1, i.e. we replicated the effect of target-distractor similarity (p<0.0001, *F* = 17.15), of position (p = 0.036, *F* = 2.51) and of orientation contrast (p = 0.012, *F* = 4.63) on letter detection probability.

#### Relation between attention allocation on the target and target detection

**Figure 8:**
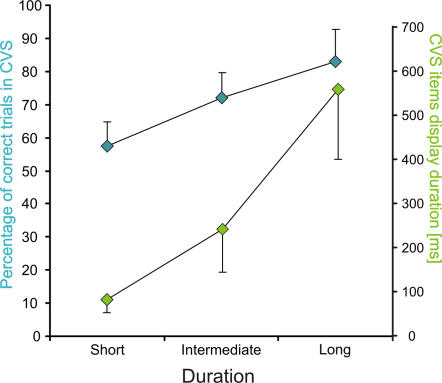
CVS performance as a function of display duration. Percentage of correct trials in CVS (cyan) and mean CVS items display duration (green) for each of the three duration conditions.

Experiment 2 was designed to allow us to determine the relationship between attention allocation and target detection since in this task, the letter detection can only occur at the very end of the search process. The relationship between the display duration and subject performance is illustrated in Figure 8. For the CVS task, the better performance associated with longer display presentation was mainly due to a decrease in FALSE ALARM rate (RM ANOVA with FALSE ALARM rate in function of CVS items display duration condition: p<0.0001, *F* = 52.74) rather than to an increase in HIT responses (RM ANOVA: p = 0.35, *F* = 1.13). This indicates that, when the display was presented for a short period of time, the subjects favored the “target-present” responses and that the opposite was true for longer display durations. This bias was estimated by the C value, based on signal detection theory. We found a significant decrease of the C value as the duration of display presentation decreased (C value × display duration, RM ANOVA: p<0.0001, *F* = 23.81). Because of this bias, in the following analysis, we used the percentage of correct trials for both “target-present” and “target-absent” trials as a measure of the performance; this measure is almost proportional to d' (R = 0.97) in the range of values used, and is more appropriate for the present analysis, given the fact that we compute its correlation to another performance value measured in percentage, thus having a more comparable distribution.

**Figure 9:**
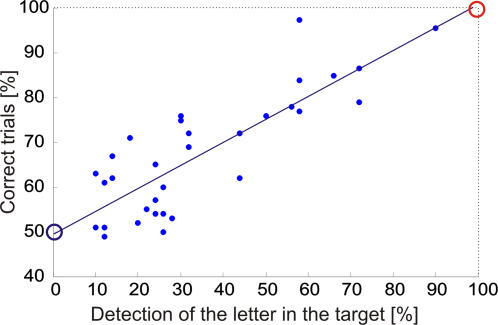
Correlation between the percentage of correct trials and percentage of detection of the letter in the target. Each dot represents data from one subject for one display duration. Note that the left extremity of the regression line (blue circle) corresponds to 50% correct responses in the CVS task and 0% of letter detection in the target and that the right extremity (red circle) corresponds to 100% correct response in the CVS task and 100% of letter detection in the target.

We found a strong correlation between the percentage of correct trials in the CVS (CORR_CVS) and the percentage of detected letters flashed inside the target (TARG_LET) (R = 0.82, p<0.0001; see Figure 9). This suggests that, whatever the display duration, the CVS performance was always proportional to the percentage of letter detected inside the target. Moreover, the regression coefficients (0.49 for the intercept and 0.51 for the slope) indicate that when the CVS task is performed at random (50% of correct trials) the letter detection rate in the target is 0%, and that a 100% performance in the CVS task is accompanied by a 100% of letter detection in the target, confirming our hypothesis. However, we must be cautious while interpreting this correlation because the difference in performance between subjects and the display duration can be considered as hidden variables. To remove the effect of these variables from the regression between CORR_CVS and TARG_LET, we computed a multiple regression with CORR_CVS as dependent variable and TARG_LET and CVS display duration as independent variables with subjects being included as dummy variables. The partial correlation between CORR_CVS and TARG_LET remained highly significant (R = 0.67, p = 0.0003). The slope of the regression was still 0.58±0.14 (b±SE) indicating that a 1% increase in letter detection performance corresponds to a .5% increase in CVS performance, like in the previous analysis. This shows that even when considering only the variations of TARG_LET and CORR_CVS which are not predicted by the display duration or the difference in performance between subjects, a comparable correlation is observed. These results suggest that the detection of the target never occurred if attention was not allocated on the target and that, when attention was allocated on the target, as shown by the letter detection, the target was always detected.

To corroborate this conclusion, we analyzed trials where the target was not detected (MISS trials). If the above conclusion is correct, in MISS trials, letters should not be detected more often inside the target than inside the distractors. Indeed, we found that in MISS trials, the percentage of detection of letters flashed inside the target was not different from the percentage of detection in distractors (one-way RM ANOVA, p = 0.59, F = 0.65; 20.10±11.01% (mean±SD), and 19.83±9.19%, 17.06±4.94% and 19.35±7.37% for level 3, 2 and 1 distractors, respectively). Since in Experiment 2, no processing of CVS items could occur after the letter detection, because the letters were always flashed during the last 50 ms of the CVS items display, this finding indicates that when the target was present but undetected, attention was not located on the target.

### Experiment 3

**Figure 10:**
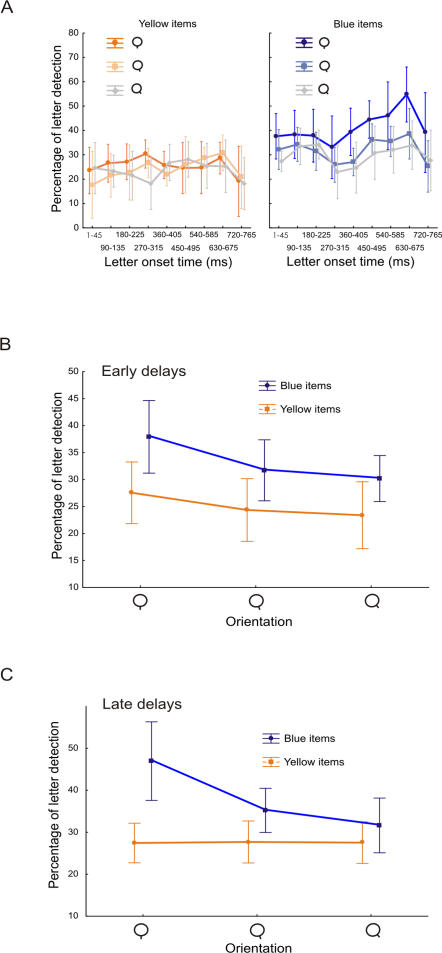
Effect of color and orientation of the CVS items on letter detection performance. A. Triple interaction orientation – color – letter onset time with letter detection performance as dependent variable. The Y-axis represents letter detection probability and the X-axis, letter-onset times with 90 ms intervals. The left and right graphs are for yellow (non target color) and blue (target color) items respectively. Color code is for orientation. The more saturated color corresponds to vertical (target orientation), the gray color corresponds to the most dissimilar distractor orientation, and the intermediate color is for the most similar distractor orientation. B. orientation – color interaction for early delays. The Y-axis represents letter detection probability, the X-axis the orientation of the items and the color code identifies the color of the item. C. orientation – color interaction for late delays. Same convention as in B.

On average, subjects reported 1.72±0.64 letters (mean±SD, n = 8) per trial. The median RT was 1332 ms with an IQI of 908 ms for “target present” trials and 1344 ms (IQI : 865 ms) for “target absent” trials. We computed a three-way RM ANOVA with color and orientation of the circles, and letter onset time as independent variables, and letter detection probability as dependent variable (see Fig. 10 A). We found that the letter detection rate increased with the onset time (*F* = 2.88, p = 0.009), except for the last onset time (765–810 ms), which was associated with a decreased letter detection rate. This last effect can probably be explained by the influence of the masks displayed at the end of each trial. The main effects of orientation (*F* = 44.88, p<0.0001) and color (*F* = 24.58, p = 0.002) were significant, confirming the results from Kim & Cave, and replicating the goal-directed salience effect we found in the feature visual search task (Experiment 1): letters displayed in items with the same color, or the same orientation as the target, were better detected.

The time – orientation and time – color interactions were not significant (p>0.1). The orientation – color interaction (*F* = 7.27, p = 0.007) was significant, showing that the orientation affected the letter detection probability for items sharing the target color only, suggesting the existence of grouping phenomena. However, the significant triple interaction (*F* = 1.82, p = 0.037, see Fig. 10 A.) indicated that this effect depended on time. While the effect of orientation for items sharing the target color clearly increased with time, the preference for the item sharing the target orientation, but with the non-target color, was only present for early letter onset times and disappeared for onset times beyond 360 ms. These very interesting effects unfortunately did not reach significance in Post-Hoc tests. As a consequence, in order to clarify the effect of early versus late delays, we computed a two-way RM ANOVA with orientation and color as independent variables for letters appearing either early (1–315 ms) or late (360 ms to 675 ms). Longer letter onset times (720–765 ms) were discarded from this analysis, because of the strong decrease in global letter detection performance at these delays. For early delays, the orientation-color interaction was not significant at all (*F* = .69, p = 0.52, see Fig. 10 B.), whereas it was significant for late delays (*F* = 26.72, p<0.0001, see Fig. 10 C.). This suggests that, at the beginning of the search, both color and orientation affect attentional guidance, and their effect are independent or combined additively, as predicted by GST. However, for longer delays, all items with the non-target color are excluded from the search process, irrespectively of their orientation, whereas the selection probability of the items that share the target color continue to depend strongly on their orientation. This finding can be regarded as evidence for the existence of grouping phenomena. We could not run the same analysis for target absent trials because the design was not balanced. Indeed, the color-orientation combination corresponding to the target never occurred.

## Discussion

In the present study, we demonstrate that, during a CVS task, very fast parallel processes guide attention towards the item with the highest saliency, as estimated from goal-directed (target-distractor similarity) and stimulus-driven (orientation contrast) factors, but also from spatial location. We also show that this guidance of attention towards the target is a prerequisite for target detection. Finally, we demonstrate a critical role of grouping phenomena in the later stage of conjunction search processes.

### Influence of goal-directed salience on attentional allocation

The finding that the letter detection probability increased as a function of target-distractor similarity indicates that goal-directed salience factors play a crucial role in attention guidance during inefficient CVS. Indeed, the fact that letters displayed inside the target and in the most resembling distractors were more likely to be detected suggests that a given goal-directed salience value, proportional to target-distractor similarity, was assigned to each CVS item. It is noteworthy that, whereas this goal-directed salience factor was feature-based (dependent on verticality), the effect we measured only relies on spatial attention, namely, the letter was detected because of its inclusion in the spotlight directed on the target location. Our results are in agreement with the existence of a guidance mechanism of spatial attention by features but cannot be interpreted in terms of location-independent global feature-based selection. In addition, we showed that this attention guidance acts very rapidly, since letters in the target were already selected preferentially in the 50 ms SOA condition.

#### Role of stimulus-driven salience in attentional allocation

The present study also demonstrates the critical influence of a stimulus-driven salience factor, the local orientation contrast, in the letter detection task. The local orientation contrast was defined as the orientation difference between a given CVS item and its two neighbors and we found that the greater this orientation difference, the higher the letter detection rate. This finding contrasts with previous studies showing that distractors with a high stimulus-driven salience have no effect on CVS performance [25], [26], a result regarded as evidence for an influence of top-down factors preventing the capture of attention by distractors. However, this discrepancy may be explained if we assume that the stimulus-driven salience factors used in those studies were irrelevant, whereas in the present task, the orientation is a target-defining feature that subjects need to process. This hypothesis about the necessity of relevance, with respect to the task at hand, of the feature defining stimulus-driven salience, is supported by the finding that when subjects were asked to report letters without performing the CVS task (see control task A), this feature-contrast effect was no longer observed.

#### Effect of spatial location on attentional allocation

The role of spatial location on attentional allocation in inefficient CVS was also evidenced in the present study. We found a higher detection rate for both the target and letters when located along the horizontal meridian. Since the stimulus eccentricity was 4°, it could be argued that this preference for stimuli located along the horizontal meridian was due to a higher visual acuity along the horizontal axis and not by an attentional effect [27], [28]. However, current knowledge on visual acuity anisotropies does not support this hypothesis. Indeed, for example, Beirne et al. [29], only found very small differences between visual acuity along the horizontal and vertical meridians for an eccentricity of 10° and this difference is probably even smaller at an eccentricity of 4°. Therefore, it is unlikely that the acuity variation could explain the large benefit in detection performance we found for letters located near the horizontal meridian. Finally, whereas Carrasco et al. [30], using a cueing paradigm, failed to find evidence that cued attention can affect the shape of the performance fields, suggesting instead an influence of visual constraints on the performance field, another study [31] has reported a performance anisotropy comparable to the one found in the present study, supporting an attentional origin for this effect. Altogether, those results suggest that the influence of spatial location that we observed in the present study is most likely due to a higher probability of allocating attention along the horizontal meridian. This bias could either be related to the probabilistic structure of the natural environment and/or correspond to a consequence of our reading and writing expertise. Finally, it is noteworthy that, in contrast to previous studies showing a better attentional resolution in the lower than in the upper visual hemifield [17], [18], we failed to find such a vertical asymmetry. However, a vertical asymmetry has been exclusively observed in crowding and tracking paradigms, suggesting that it may not be an invariant feature of attention which can be generalized to all CVS tasks.

Although the influence of both spatial location and orientation contrast on CVS performance is unquestionable in the present study, the behavioral benefit of these factors remains puzzling. Indeed, whereas goal-directed salience factors are directly related to the cognitive context of the task at hand, local orientation contrast and spatial location are ineffective to help locating the target. In Guided Search Theory, Wolfe and colleagues [5] suggested that stimulus-driven factors are automatically included into the activation map, and hence, influence attention guidance irrespective of the task at hand. The behavioral significance of these salience factors could be revealed in more ecological situations where it may be pertinent to pay attention to high-contrast objects and to objects located along the horizontal meridian. The role of these factors in the construction of the salience map could thus be deeply rooted into the probabilistic structure of the visual world.

#### Relationship between attentional selection and target detection

Regarding the question as to whether target selection follows or precedes target detection, we showed in Experiment 2, that the correlation between target detection rate and letter detection rate in the target was perfectly linear. Moreover, the regression showed that minimal (chance level) and maximal (100%) performance in target detection corresponded to 0% and 100% performance in letter detection rate in the target. This suggests that attentional selection is a prerequisite for target detection. Indeed, if target selection followed target detection, and that a better detection of the letter in the target was a consequence of a post-detection shift of attention on the target, we should have found instances, for short display duration, where the target was detected but the display ended before the attentional shift could occur. In this case, the subjects would have failed to detect the letter flashed inside the target and the regression line represented in Figure 9 should have been shifted upward. In addition, the finding that the goal-directed salience exerted a strong effect on attention allocation in trials where the target was missed and, in Experiment 1, when no target was present, is also in contradiction with the hypothesis that attention selection only follows target detection.

#### Grouping phenomena

In Experiment 3, we found that the goal-directed effects of orientation and color are initially combined in a global activation map but that later on, during the CVS processing, the interaction becomes more complex: non-target color items are excluded from the search process, irrespectively of their orientation. We interpret these results as supporting the existence of a two-stage process: first, salience is initially computed from the addition of feature maps, as predicted by Guided Search Theory, and then grouping effects are involved at a later stage, after a 360 ms delay in our Experiment.

This long delay could appear surprising, given the fact that perceptual grouping has been reported for very short display durations [32]. However, it has been shown that different mechanisms of perceptual grouping occur at different stages of visual processing: while early grouping depends on retinal color similarity, surface color similarity would be involved for grouping at long durations [33]. It is likely that this latter mechanism could be responsible for the grouping effect we observed in our task. Several previous studies argued for a central role of grouping processes in conjunction CVS [15], [19]–[22]. In the present study, we showed for the first time that such grouping phenomena are involved at a late stage in the visual search process.

## Conclusions

The present results are consistent with the existence of a neuronal salience map [34] where different hills of activity, one for each stimulus position, are weighted by both goal-directed [35] and stimulus-driven salience factors, and by spatial location. This salience map is used subsequently to guide attention. The existence of parallel mechanisms of attentional guidance in CVS could explain the apparent discrepancy between the slope values of the regression line between RT and item number (“set-size effect”), and the minimum time required to shift attention [36]. Indeed, this preattentive salience computation could make CVS more efficient by reducing the number of attentional shifts needed to find the target, leading therefore to a shorter processing time for each additional item. Other phenomena, like perceptual grouping, are also involved later, to increase further the efficiency of the search process, by selecting a whole group of pertinent items at once.

Additionally, the present results show that target detection always follows attentional selection. Consequently, this suggests that target detection never occurs in the absence of attentional selection. An interesting study has already addressed this question in dual tasks involving a central and a peripheral concurrent discrimination tasks [37]. Performance in the peripheral discrimination task was compared when performed together with the central discrimination task and when performed alone. In the dual task condition, attention was supposed to be focused on the central discrimination task, whereas in the single task condition, attention was able to select the peripheral target. This study showed that the detection performance in the peripheral task when attention was focused on the central task depended on the nature of the stimuli used: some discrimination tasks, even very complex, were left almost unaltered by the retrieval of attention, while other tasks, seemingly simple, were much impaired in the absence of attention. Along the same line, Joseph et al. [38] asked their subjects to perform a pop-out task and were able to demonstrate that a concurrent attention-demanding task (rapid serial visual presentation task) strongly altered performance in the CVS task, suggesting that even pop-out CVS tasks depend on the availability of attention. However, Van Rullen et al. [39] showed that targets that do not trigger pop-out could be discriminated from distractors with attention occupied elsewhere and that, conversely, certain pop out CVS tasks need undivided attention to be performed. Altogether, these different studies suggest that the dichotomy between attention-demanding and not attention-demanding CVS tasks might differ from the classic efficient (pop-out) versus inefficient CVS tasks and this question would require further experiments.
